# Geraniin Alleviates Mouse Laser‐Induced Choroidal Neovascularisation by Inhibiting Choroidal Endothelial Cell ACE2/Ang‐(1–7)/MasR/IL‐10 Pathway

**DOI:** 10.1111/jcmm.70228

**Published:** 2024-12-02

**Authors:** Hongyi Lu, Qi Cai, Lele Li, Jiayi Gu, Yuting Zhang, Haotian Sun, Hui Su, Lei Song

**Affiliations:** ^1^ Department of Pediatrics Nantong First People's Hospital (The Second Affiliated Hospital of Nantong University) Nantong Jiangsu China; ^2^ Department of Ophthalmology Nantong First People's Hospital (The Second Affiliated Hospital of Nantong University) Nantong Jiangsu China; ^3^ Department of Ophthalmology Lixiang Eye Hospital of Soochow University Suzhou Jiangsu China

**Keywords:** choroidal endothelial cells, choroidal neovascularisation, Geraniin, interleukin‐10

## Abstract

Anti‐vascular endothelial growth factor (VEGF) drugs suppress choroidal neovascularisation (CNV), thus improving vision. However, some patients may have a poor response or develop resistance to anti‐VEGF drugs. Geraniin (GE), a polyphenol isolated from an herb called *Phyllanthus amarus* , possesses anti‐angiogenic properties. This study aimed to explore the mechanism of action of GE in CNV. GE was found to activate the angiotensin‐converting enzyme 2 (ACE2)/angiotensin 1–7 (Ang‐[1–7])/MAS1 proto‐oncogene, G protein‐coupled receptor (MasR)/interleukin‐10 (IL‐10) pathway in hypoxic human choroidal endothelial cells (HCECs) in vitro and mouse models of laser‐induced CNV in vivo. Activation of the ACE2/Ang‐(1–7)/MasR/IL‐10 pathway by GE attenuated the proliferative, migratory, and tube‐forming abilities of hypoxic HCECs and prevented the development of CNV in mice. Notably, GE did not cause ocular or systemic toxicity in mice with CNV. These findings suggest that GE alleviates CNV by activating the ACE2/Ang‐(1–7)/MasR/IL‐10 pathway in choroidal endothelial cells (CECs).

## 1 Introduction

Choroidal neovascularisation (CNV) is characterised by the abnormal intravasation of choroidal vasculature into the retinal epithelium or subretinal tissue [1]. The abnormal vessels leak clear fluid or blood into the macula, leading to severe central vision loss [2]. CNV is associated with multiple ocular diseases, including pathologic myopia [3], central serous chorioretinopathy [4], and age‐related macular degeneration [5]. Owing to the important role of vascular endothelial growth factor (VEGF) in the development of CNV [6], anti‐VEGF drugs are used as the first‐line treatment agents for CNV [7]. However, these drugs have several limitations, including the requirement for repeat intravitreal injections, which imposes a substantial economic and psychological burden on patients [8]; development of tolerance [9]; and risk of adverse reactions such as retinal detachment, endophthalmitis, and macular atrophy [10]. Therefore, developing alternative therapeutic strategies for CNV is necessary.

Geraniin (GE), a polyphenol isolated from the herbal plant *Phyllanthus amarus* [11], possesses antioxidative and anti‐inflammatory properties [12, 13]. A study showed that treatment with 0.2% w/w GE enhanced angiogenesis in wound tissues from rat excision wound models [14], indicating that GE exhibits pro‐angiogenic activity. However, the role and mechanism of action of GE in CNV remain unclear.

Angiotensin‐converting enzyme 2 (ACE2), a member of the renin–angiotensin system (RAS), plays an anti‐angiogenic role in breast cancer [15]. The hepatic mRNA expression of ACE2 is negatively correlated with that of CD34, an angiogenesis marker, in hepatocellular carcinoma (HCC) [16]. In addition, ACE2 suppresses angiogenesis and inhibits the expression of VEGF in non‐small cell lung cancer (NSCLC) [17]. GE has a high affinity for ACE2 [18] expressed on choroidal endothelial cells (CECs) [19]. ACE2 directly hydrolyses angiotensin II (Ang II) to yield angiotensin 1–7 (Ang‐[1–7]), which activates downstream MAS1 proto‐oncogene, G protein‐coupled receptor (MasR). Activation of the ACE2/Ang‐(1–7)/MasR pathway increases the production of interleukin‐10 (IL‐10) during inflammation [20]. As a cytokine, IL‐10 not only modulates the inflammatory response [21] but also acts as an anti‐angiogenic factor [22, 23].

Herein, we investigated the mechanism of action of GE in CNV using hypoxia‐stimulated human choroidal endothelial cells (HCECs) in vitro and mouse models of laser‐induced CNV in vivo.

### 1.1 Cell Culture and Treatment

HCECs (CP‐H092, Procell, China) were cultured in Dulbecco's modified Eagle medium/Ham's F12 medium (DMEM/F‐12) (21041025, Gibco, USA) supplemented with 10% fetal bovine serum (FBS) (10099141, Gibco) and 1% antibiotic–antimycotic solution (15240062, Gibco) [24]. ARPE‐19 cells (CRL‐2302, ATCC, USA) were cultured in DMEM/F12 supplemented with GlutaMAX (35050061, Gibco), 10% FBS, and 1% antibiotic–antimycotic solution. The human Müller cell line Moorfields/Institute of Ophthalmology‐Müller 1 (MIO‐M1) was obtained from the UCL Institute of Ophthalmology, London, UK [25]. MIO‐M1 cells were cultured in low‐glucose DMEM (D5921, Sigma‐Aldrich, USA) supplemented with 10% FBS, GlutaMAX, and 1% antibiotic–antimycotic solution. All cells were maintained at 37°C in a humified incubator with 5% CO_2_. HCECs were treated with GE (HY‐N0472, MedChemExpress, USA), the ACE inhibitor MLN‐4760 (100 nM for 24 h) (HY‐19414, MedChemExpress), the Ang‐(1–7) antagonist D‐Pro7‐Ang‐(1–7) (1 μM for 24 h) (50–194‐6749, Thermo Fisher Scientific), the MasR antagonist A‐779 (1 μM for 24 h) (HY‐P0216, MedChemExpress), or neutralising anti‐IL‐10 antibody (2 μg for 24 h) (MAB217, R&D Systems, USA).

### 1.2 Western Blotting

Western blotting was performed as described previously [26]. The primary antibodies used for western blotting included rabbit ACE2 (1:2000) (MA5‐32307, Invitrogen, USA), rabbit MasR (1:1000) (ab235914, Abcam, USA), mouse IL‐10 (1:2000) (60269‐1‐Ig, Proteintech, USA), mouse GAPDH (1:100000) (60004‐1‐Ig, Proteintech), rabbit β‐tubulin (1:1000) (2146, Cell Signalling Technology, USA), and mouse Na/K/ATPase (1:1000) (sc‐514614, Santa Cruz Biotechnology, USA). The secondary antibodies used for western blotting included horseradish peroxidase (HRP)‐conjugated goat anti‐rabbit IgG (1:20000) (31460, Invitrogen) and HRP‐conjugated goat anti‐mouse IgG (1:10000) (31430, Invitrogen).

### 1.3 Isolation of the Plasma Membrane

The plasma membrane was isolated from HCECs and mouse retina–retinal pigment epithelium (RPE)–choroid tissues using a plasma membrane isolation kit (ab284937, Abcam, USA) according to the manufacturer's instructions.

### 1.4 Quantitative Reverse Transcription Polymerase Chain Reaction

Quantitative reverse transcription polymerase chain reaction (qRT‐PCR) was performed as described previously [27]. The primer sequences used for PCR are as follows: ACE2 forward, 5’‐ACACTGATGATGTTCAGACCTCC‐3′; ACE2 reverse, 5′‐ GCTCTCTCCTTGGCCATGTT‐3′; Na/K/ATPase forward, 5’‐CTCTGATTCTCCAGCGACAGG‐3′; Na/K/ATPase reverse, 5’‐TAATCCCCGGCTCAAGTCTG‐3′.

### 1.5 Assessment of the Enzymatic Activity of ACE2

The activity of ACE2 in cell lysates and tissues was measured using a fluorometric ACE2 activity assay kit (ab273297, Abcam) according to the manufacturer's instructions. The measurements were performed in black plates (3916, Corning, USA) with a total volume of 100 mL (including both lysates and ACE2 substrate). After HCECs were exposed to different treatments for 24 h, they were lysed and used to assess the activity of ACE2. Similarly, retina–RPE–choroid tissues harvested from mice were used to assess the activity of ACE2. Fluorescence intensity was measured at an excitation wavelength of 320 nm and an emission wavelength of 420 nm on a multiplate reader (SpectraMax 5, USA).

### 1.6 Enzyme‐Linked Immunosorbent Assay

The levels of Ang II and Ang‐(1–7) proteins in HCECs and those of the IL‐10 protein in the culture supernatants of HCECs were assessed using a human Ang II ELISA kit (RAB0010, Sigma‐Aldrich), human Ang‐(1–7) ELISA kit (abx153636, Abbexa, USA), and human IL‐10 ELISA kit (88‐7106‐22, Invitrogen), respectively. The levels of Ang‐(1–7) and IL‐10 proteins in mouse retina–RPE–choroid tissues were assessed using a mouse Ang‐(1–7) ELISA kit (NBP2‐69079, Novus Biologicals, USA) and mouse IL‐10 ELISA kit (ab108870, Abcam), respectively. All enzyme‐linked immunosorbent assay (ELISA) kits were used according to the manufacturers' instructions.

### 1.7 5‐Ethyl‐2′‐Deoxyuridine Incorporation Assay

The proliferative ability of HCECs was evaluated using a Click‐iT EdU cell proliferation imaging kit with Alexa Fluor 555 dye (C10338, Invitrogen) according to the manufacturer's instructions. The 5‐ethyl‐2′‐deoxyuridine (EdU) incorporation rate was calculated as the ratio of the number of EdU‐positive cells (red) to the number of total DAPI‐positive cells (blue).

### 1.8 Transwell Assay

Transwell assay was performed using 12‐mm Transwell chambers with polycarbonate membrane inserts with 0.4‐μm pores (3401, Corning, USA). HCECs in serum‐free DMEM/F‐12 were seeded in the upper chamber, whereas DMEM/F‐12 supplemented with 10% FBS was added to the lower chamber. After 24 h of incubation, the migrated cells were fixed, stained with crystal violet, and counted in 10 random fields at 100× magnification under an inverted light microscope.

### 1.9 Tube Formation Assay

A 96‐well plate was coated with growth factor‐reduced Matrigel (CLS356231, Corning) and incubated at 37°C for 30 min. HCECs were seeded in the plate at a density of 2 × 10^4^ cells/well and cultured for 24 h. Images were captured using a light microscope, and the extent of tube formation was quantified using the ImageJ software.

### 1.10 Establishment of a Mouse Model of Laser‐Induced CNV

Male C57BL/6J mice (age, 6–8 weeks) were purchased from the Laboratory Animal Center of Nantong University (Jiangsu, China). The mice were maintained on a 12‐h light/12‐h dark cycle at 22°C ± 2°C. Mouse models of laser‐induced CNV were established as described previously [28]. The mice were divided into the following groups: normal, CNV 7 d, CNV 7 d + GE (5 μM), CNV 7 d + GE (10 μM), CNV 7 d + GE (20 μM), CNV 7 d + GE + MLN‐4760 (10 nM), CNV 7 d + GE + D‐Pro7‐Ang‐(1–7) (100 nM), CNV 7 d + GE + A‐779 (100 nM), and CNV 7 d + GE + neutralising anti‐IL‐10 antibody (MA5‐23796, Thermo Fisher Scientific, USA; 1 μg). GE, MLN‐4760, D‐Pro7‐Ang‐(1–7), A‐779, and neutralising anti‐IL‐10 antibody were administered intravitreally at a volume of 1 μL one time on the day of laser irradiation. All experiments involving animals were approved by the Animal Ethics Committee of Nantong University.

### 1.11 Fundus Fluorescein Angiography (FFA) and Indocyanine Green Angiography (ICGA)

On day 7 after the laser photocoagulation, in vivo FFA and ICGA of the CNV lesion were performed using commercial retinal angiography system (Heidelberg retinal angiography system; Heidelberg Retinal Engineering, Germany). Under systemic anaesthesia and pupil dilation, a mixture of 10 mg of fluorescein sodium (00065009265, Alcon, USA) and 0.15 mg of indocyanine green (#1340009, Merck, USA) was administered intraperitoneally. Late‐phase FFA and ICGA images were captured. Leaky areas from CNV were calculated as total measured hyperfluorescent areas in FFA images divided by the total measured CNV area in ICGA images. ImageJ was used to quantify the vascular leakage of CNV lesions. Moreover, fluorescein leakage was also graded using previously established criteria: 0 (not leaky), faint hyperfluorescence or mottled fluorescence without leakage; 1 (questionable leakage), hyperfluorescent lesion without progressive increase in size or intensity; 2a (leaky), hyperfluorescence increasing in intensity but not in size; 2b (pathologically significant leakage), hyperfluorescence increasing in both intensity and in size [29].

### 1.12 Preparation of Choroidal Flat Mounts and Quantification of CNV

On day 7 after laser photocoagulation, the eyes of all mice were enucleated and fixed in 4% paraformaldehyde (PFA) for 1 h. The neural retina was peeled away from choroid/RPE tissue, and four radial incisions were made in the isolated choroid/RPE/sclera tissue. The processed choroid/RPE/sclera tissues were placed in a 24‐well culture plate and permeabilised with 0.5% Triton X‐100 for 12 h at 4°C. The tissues were washed with PBS and incubated with Alexa Fluor 488‐conjugated *Griffonia simplicifolia* isolectin B4 (IB4; I21411, Invitrogen) and Alexa Fluor 594‐conjugated collagen IV antibodies (NB120‐6586AF594, Novus Biologicals). The culture plate was wrapped with foil and incubated on a shaker (100 rpm) for 12 h at 37°C. Subsequently, the tissues were washed with PBS and stained with 4′,6‐diamidino‐2‐phenylindole (DAPI) (D1306, Invitrogen; 1:1000) for 5 min. The stained tissues were mounted on glass slides and covered with a cover slip. Images were captured using a confocal laser scanning microscope and analysed using the Volocity 3D image software (PerkinElmer).

### 1.13 Haematoxylin–Eosin Staining

After the mice were sacrificed, their eyeballs, hearts, livers, spleens, lungs, and kidneys were harvested and fixed in 4% PFA overnight at 4°C. The following day, the tissues were routinely dehydrated and embedded in Scigen Tissue‐Plus optimal cutting temperature (O.C.T) compound (25608‐930, Fisher Scientific, USA) for cryosectioning. The optic nerve parallel to the sagittal plane at the laser irradiation site was preserved. After the tissues were serially cut into 6‐μm‐thick sections, the sections were stained with haematoxylin–eosin (HE) and imaged using a light microscope. For retinal tissue sections, the ratio of the distance from the ganglion cell layer to the outer edge of the inner nuclear layer (A) to the distance from the ganglion cell layer to the outer edge of the outer nuclear layer (B) was quantified using the ImageJ software [30].

### 1.14 Terminal Deoxynucleotidyl Transferase dUTP Nick End Labelling Assay

Terminal deoxynucleotidyl transferase dUTP nick end labelling (TUNEL) assay was performed on paraffin‐embedded mouse eye sections using an in situ cell death detection kit (11684795910, Roche, Germany) according to the manufacturer's guidelines. DAPI was used to counterstain nuclei, and images were captured using a fluorescence microscope.

### 1.15 Statistical Analysis

All data were expressed as the mean ± SEM. Differences between groups were analysed using the Student's *t*‐test, whereas those among three or more groups were analysed using one‐way ANOVA followed by the Bonferroni post hoc test. The SPSS Statistics (version 20.0) software was used for statistical analysis. A *p*‐value of < 0.05 was considered statistically significant.

## 2 Results

### 2.1 GE‐Activated ACE2 in Hypoxic HCECs

**FIGURE 1:**
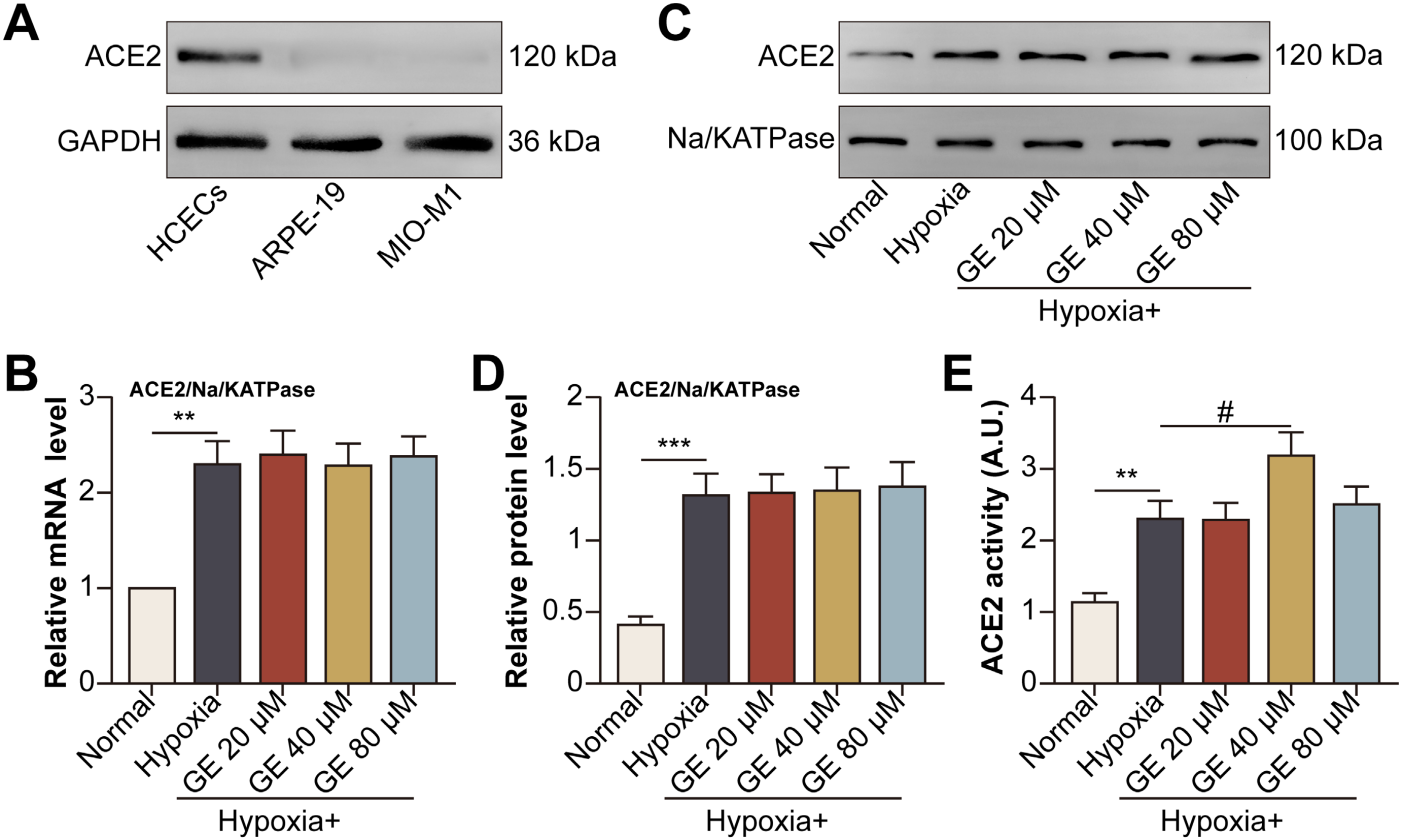
GE‐activated ACE2 in hypoxic HCECs. (A) The protein expression of ACE2 was detected in HCECs, ARPE‐19, and MIO‐M1 cells under normoxic conditions after 24 h of culture. HCECs were divided into normal, hypoxia, hypoxia + GE (20 μM for 24 h), hypoxia + GE (40 μM for 24 h), and hypoxia + GE (80 μM for 24 h) groups. (B) The mRNA expression of ACE2 on the plasma membrane of HCECs was detected via qRT‐PCR. (C) The protein expression of ACE2 on the plasma membrane of HCECs was detected via Western blotting. (D) Relative protein expression of ACE2. (E) Enzymatic activity of ACE on the plasma membrane of HCECs. All data are expressed as the area under the kinetic activity curves (**p < 0.01; ***p < 0.001 versus the normal group; ^#^ *p* < 0.05 versus the hypoxia group).

Given that ACE2 was expressed in HCECs but not in ARPE‐19 or MIO‐M1 cells (Figure 1A), HCECs were selected for subsequent experiments. Treatment with GE at different doses did not affect the mRNA (Figure 1B) or protein (Figure 1C,D) expression of ACE2 on the plasma membrane of hypoxia‐induced HCECs. However, treatment with 40‐μM GE inhibited the hypoxia‐induced enzymatic activity of ACE2 on the plasma membrane of HCECs (Figure 1E).

### 2.2 GE‐Activated ACE2 Catalysed the Conversion of Ang‐II to Ang‐(1–7) in Hypoxic HCECs

**FIGURE 2:**
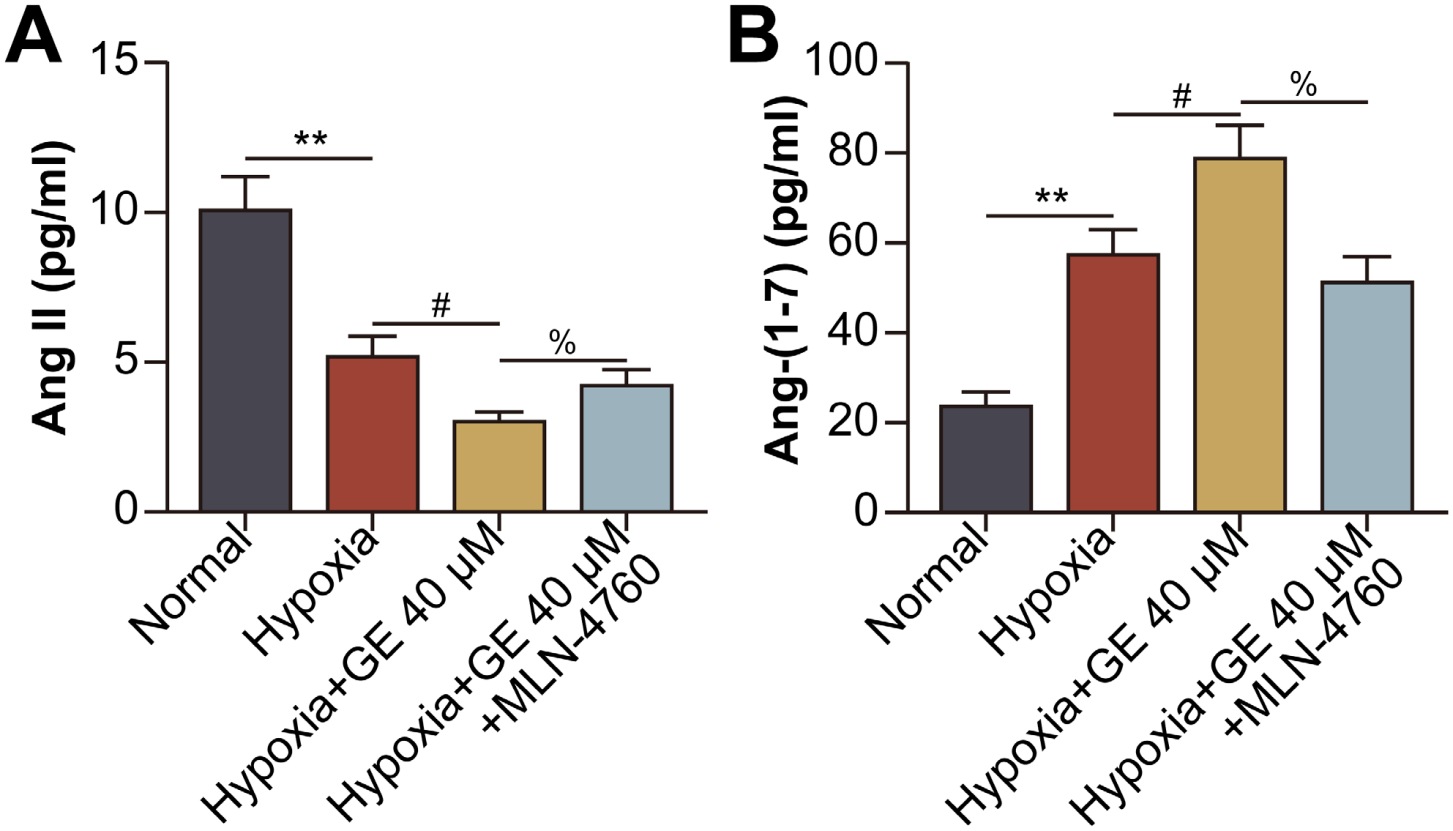
GE‐activated ACE2 catalysed the conversion of Ang‐II to Ang‐(1–7) in hypoxic HCECs. HCECs were divided into normal, hypoxia, hypoxia + GE (40 μM for 24 h), and hypoxia + GE + MLN‐4760 (100 nM for 24 h) groups. (A) The protein expression of Ang II in HCECs was detected via ELISA. (B) The protein expression of Ang‐(1–7) in HCECs was detected via ELISA (**p < 0.01 vs. the normal group; ^#^, *p* < 0.05 vs. the hypoxia group; ^%^, *p* < 0.05 vs. the hypoxia + GE group).

The expression of Ang II in HCECs decreased upon induction of hypoxia and treatment with GE but increased upon co‐treatment with GE and the ACE2 inhibitor MLN‐4760 (Figure 2A). However, changes in the expression of Ang‐(1–7), the product of ACE2, showed the opposite trend (Figure 2B).

### 2.3 Ang‐(1–7) Catalysed by GE‐Activated ACE2 Activated Downstream MasR

**FIGURE 3:**
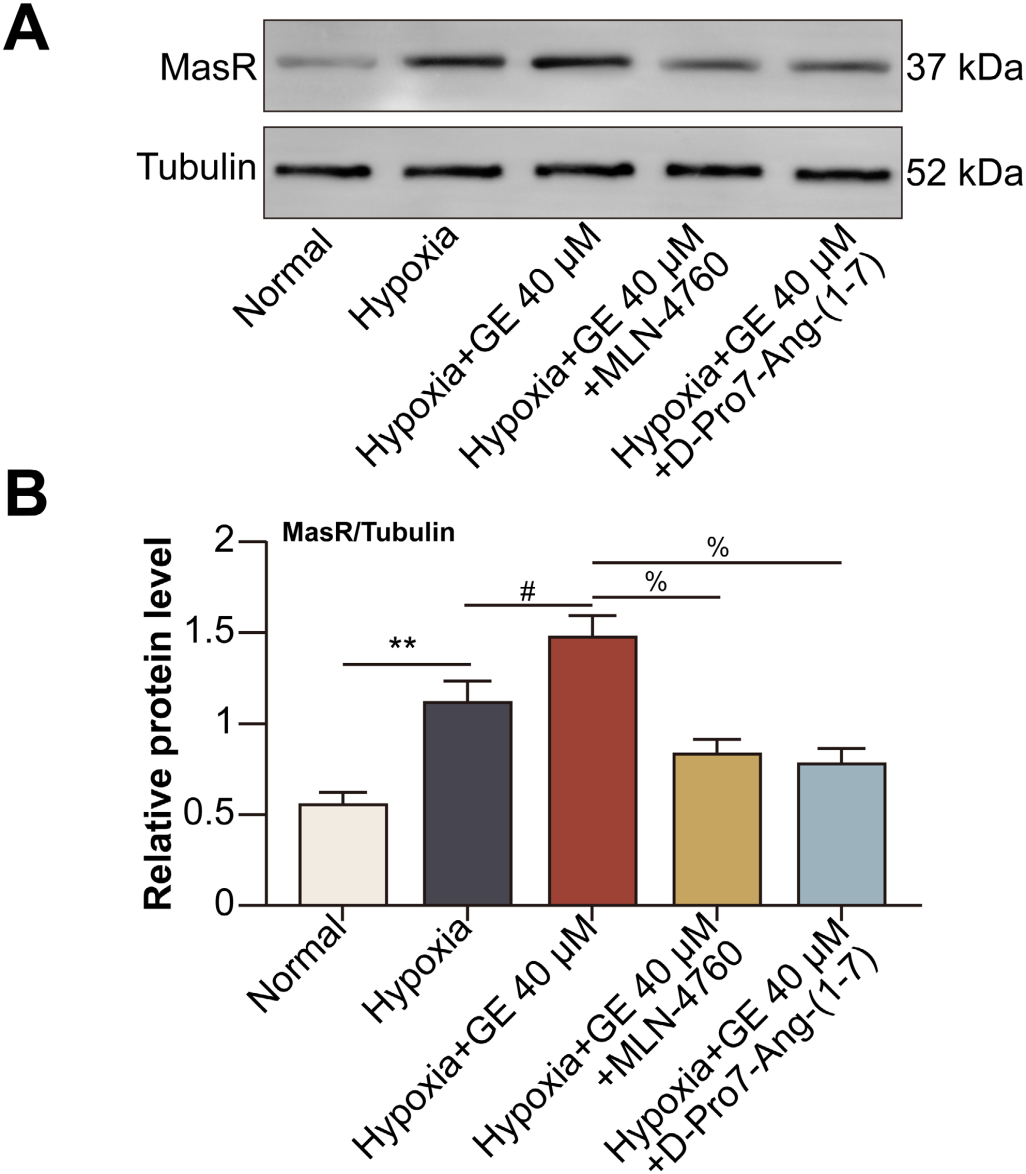
Ang‐(1–7) catalysed by GE‐activated ACE2 activated downstream MasR. HCECs were divided into normal, hypoxia, hypoxia + GE (40 μM for 24 h), hypoxia + GE + MLN‐4760, and hypoxia + GE + D‐Pro7‐Ang‐(1–7) (1 μM for 24 h) groups. (A) The protein expression of MasR in HCECs was detected via western blotting. (B) Relative protein expression of MasR (**p < 0.01 vs. the normal group; ^#^ *p* < 0.05 vs. the hypoxia group; ^%^ *p* < 0.05 vs. the hypoxia + GE group).

Treatment with GE enhanced the hypoxia‐induced expression of MasR in HCECs, whereas treatment with MLN‐4760 or the Ang‐(1–7) antagonist D‐Pro7‐Ang‐(1–7) decreased the expression of MasR in hypoxic HCECs (Figure 3A,B).

Activation of the ACE2/Ang‐(1–7)/MasR pathway by GE promoted the production and release of IL‐10 from hypoxic HCECs.

**FIGURE 4:**
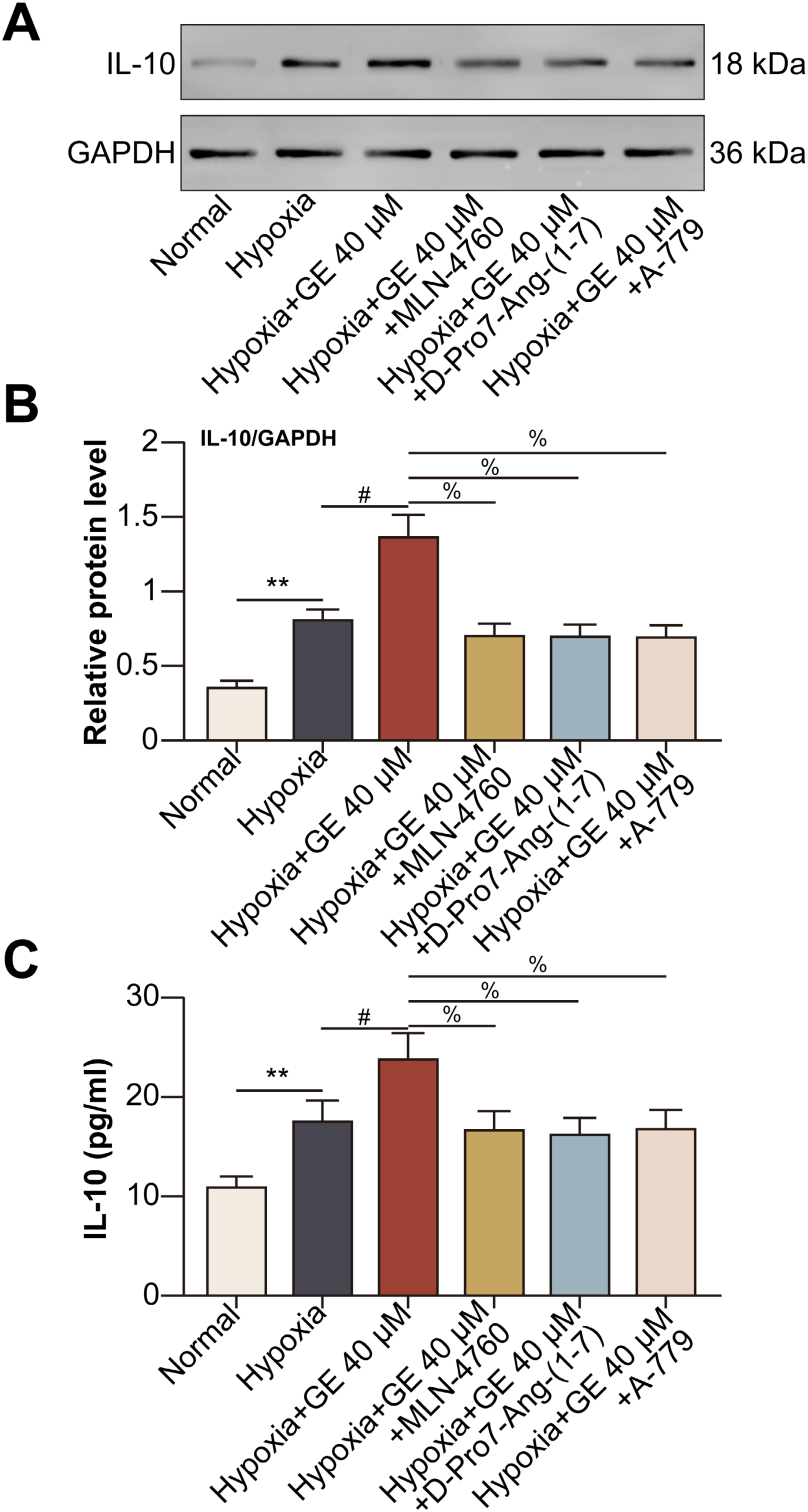
Activation of the ACE2/Ang‐(1–7)/MasR pathway by GE promoted the production and release of IL‐10 from hypoxic HCECs. HCECs were divided into normal, hypoxia, hypoxia + GE, hypoxia + GE + MLN‐4760, hypoxia + GE + D‐Pro7‐Ang‐(1–7), and hypoxia + GE + A‐779 (1 μM for 24 h) groups. (A) The protein expression of IL‐10 in HCECs was detected via Western blotting. (B) Relative protein expression of IL‐10. C IL‐10 levels in the culture supernatants of HCECs were assessed via ELISA (**p < 0.01 vs. the normal group; ^#^ *p* < 0.05 vs. the hypoxia group; ^%^ *p* < 0.05 vs. the hypoxia + GE group).

Treatment with GE increased the hypoxia‐induced expression of IL‐10 in HCECs, whereas treatment with MLN‐4760, D‐Pro7‐Ang‐(1–7), or the MasR antagonist A‐779 decreased the expression of IL‐10 in hypoxic HCECs (Figure 4A,B). Changes in IL‐10 expression in the culture supernatants of HCECs were consistent with those in HCECs (Figure 4C).

Activation of the ACE2/Ang‐(1–7)/MasR/IL‐10 pathway by GE reduced the proliferative, migratory, and tube‐forming abilities of hypoxia‐induced HCECs.

**FIGURE 5:**
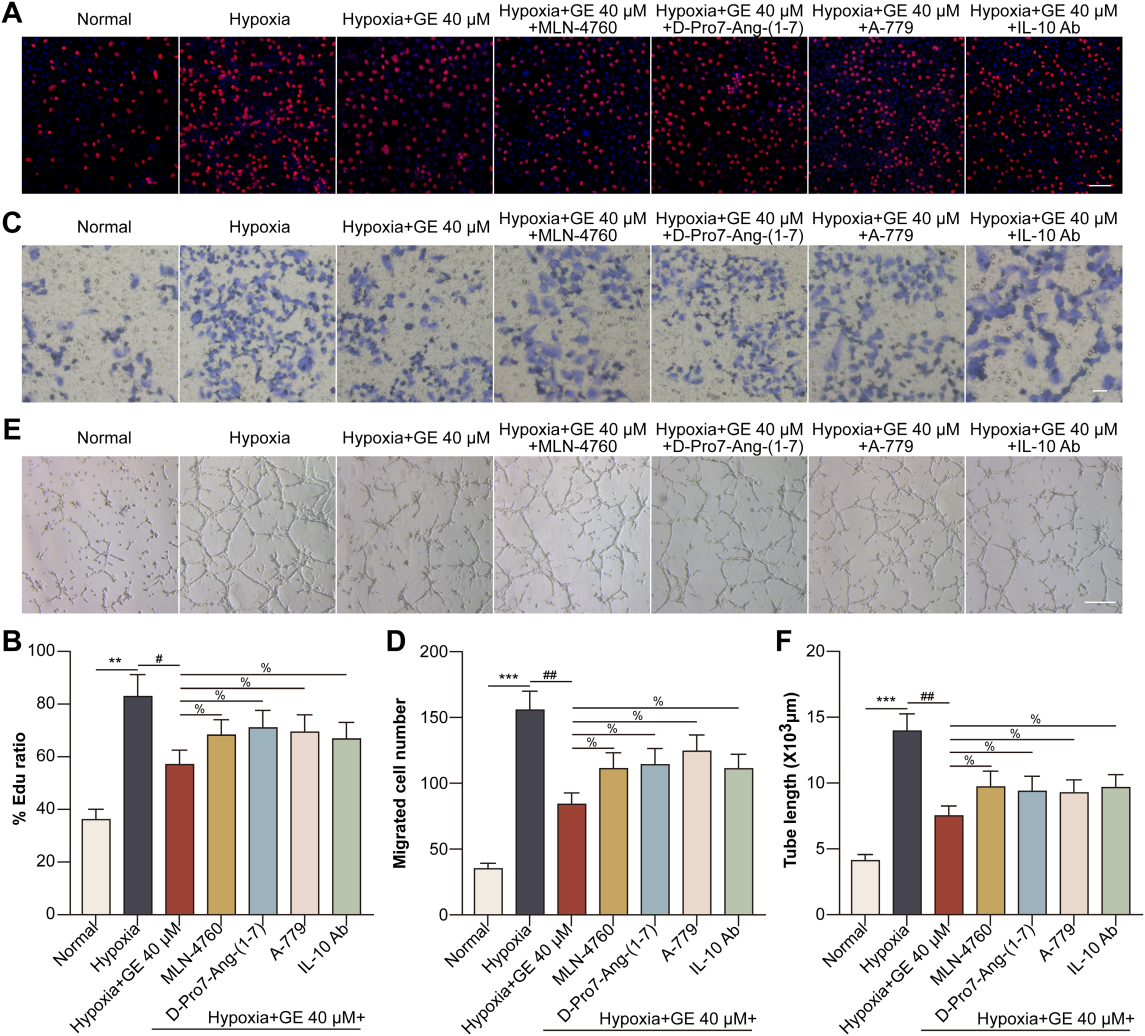
Activation of the ACE2/Ang‐(1–7)/MasR/IL‐10 pathway by GE suppressed the hypoxia‐induced proliferative, migratory, and tube‐forming abilities of HCECs. HCECs were divided into normal, hypoxia, hypoxia + GE, hypoxia + GE + MLN‐4760, hypoxia + GE + D‐Pro7‐Ang‐(1–7), hypoxia + GE + A‐779, and hypoxia + GE + neutralising anti‐IL‐10 antibody (2 μg for 24 h) groups. (A) EdU incorporation assay was performed to assess the proliferative ability of HCECs. (B) Ratio of the number of EdU‐positive cells to the number of DAPI‐positive cells. (C) Transwell assay was performed to assess the migratory ability of HCECs. (D) Number of migrated HCECs. (E) Tube formation assay was performed to assess the tube‐forming ability of HCECs. (F) Length of the tubes formed (all scale bars = 50 μm) (**p < 0.01 vs. the normal group; ^#^ *p* < 0.05; ^##^ *p* < 0.01 vs. the hypoxia group; ^%^ *p* < 0.05 vs. the hypoxia + GE group).

Treatment with GE attenuated the hypoxia‐induced proliferative, migratory, and tube‐forming abilities of HCECs. However, treatment with MLN‐4760, D‐Pro7‐Ang‐(1–7), A‐779, or neutralising anti‐IL‐10 antibody partially counteracted the inhibitory effects of GE on the proliferation (Figure 5A,B), migration (Figure 5C,D), and tube formation (Figure 5E,F) of hypoxic HCECs.

### 2.4 GE Activated the ACE2/Ang‐(1–7)/MasR/IL‐10 Pathway in Mice With Laser‐Induced CNV

**FIGURE 6:**
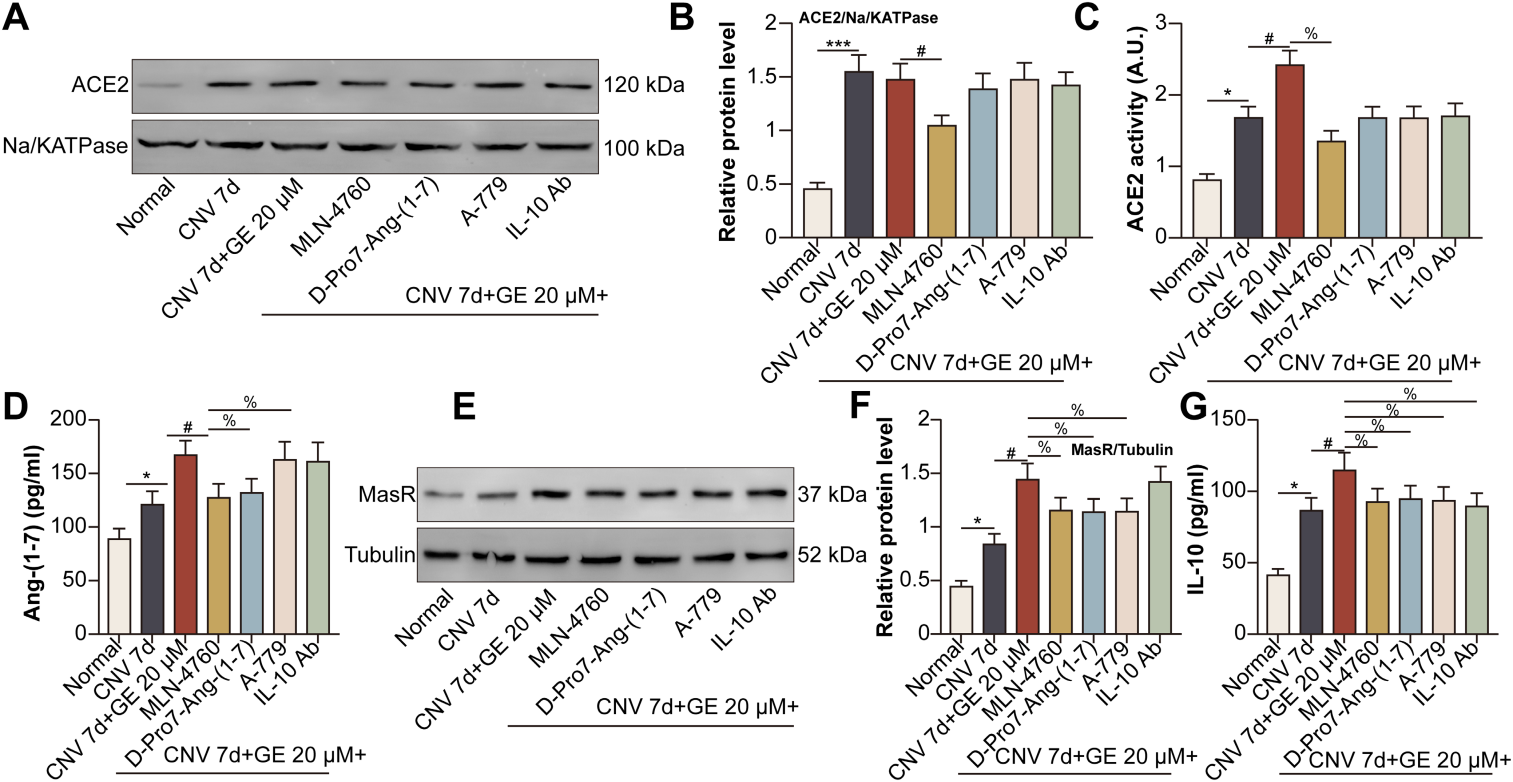
GE activated the ACE2/Ang‐(1–7)/MasR/IL‐10 pathway in mice with laser‐induced CNV. Mice were divided into normal, CNV 7 d, CNV 7 d + GE (20 μM), CNV 7 d + GE + MLN‐4760 (10 nM), CNV 7 d + GE + D‐Pro7‐Ang‐(1–7) (100 nM), CNV 7 d + GE + A‐779 (100 nM), and CNV 7 d + GE + neutralising anti‐IL‐10 antibody (1 μg) groups. (A) The protein expression of ACE2 in the plasma membrane of mouse retina–RPE–choroid tissues was detected via western blotting. (B) Relative protein expression of ACE2. (C) The activity of ACE2 in the plasma membrane of mouse retina–RPE–choroid tissues was measured using an ACE2 activity detection kit. (D) The protein expression of Ang‐(1–7) in mouse retina–RPE–choroid tissues was detected via ELISA. (E) The protein expression of MasR in mouse retina–RPE–choroid tissues was detected via western blotting. (F) Relative protein expression of MasR. (G) The protein expression of IL‐10 in mouse retina–RPE–choroid tissues was detected via ELISA (**p < 0.01 vs. the normal group; ^#^ *p* < 0.05 vs. the CNV 7 d group; ^%^ *p* < 0.05 vs. the CNV 7 d + GE group).

To choose the optimal concentration of GE, FFA and ICGA following intravitreal injection of GE at different concentrations for CNV 7 d mice were performed. The results displayed that GE at 5 μM had no influence on CNV leakage and area. Meanwhile, GE at the concentration of 20 μM, decreased CNV leakage (Figure S1A,B) and area (Figure S1C,D) more effectively than those of GE at 10 μM. For this reason, the concentration of 20 μM for GE was determined for subsequent mouse experiments. The expression of ACE2 in the plasma membrane of retina–RPE–choroid tissues was higher in the CNV 7 d group than in the normal group. The expression of ACE2 in mice with CNV remained unaltered upon treatment with GE but decreased upon treatment with MLN‐4760 (Figure 6A,B). GE enhanced the CNV‐induced enzymatic activity of ACE2 in the plasma membrane of retina–RPE–choroid tissues, whereas MLN‐4760 decreased the activity of ACE2 in GE‐treated mice with CNV (Figure 6C). GE increased the expression of Ang‐(1–7) in retina–RPE–choroid tissues from mice with CNV, whereas MLN‐4760 or D‐Pro7‐Ang‐(1–7) counteracted this effect (Figure 6D). GE increased the expression of MasR in retina–RPE–choroid tissues from mice with CNV, whereas MLN‐4760, D‐Pro7‐Ang‐(1–7), or A‐779 reversed this change (Figure 6E,F). In addition, GE enhanced the production of IL‐10 in retina–RPE–choroid tissues from mice with CNV; however, MLN‐4760, D‐Pro7‐Ang‐(1–7), A‐779, or neutralising anti‐IL‐10 antibody decreased the production of IL‐10 in GE‐treated mice with CNV (Figure 6G).

GE prevented the development of CNV by activating the ACE2/Ang‐(1–7)/MasR/IL‐10 pathway in mice.

**FIGURE 7:**
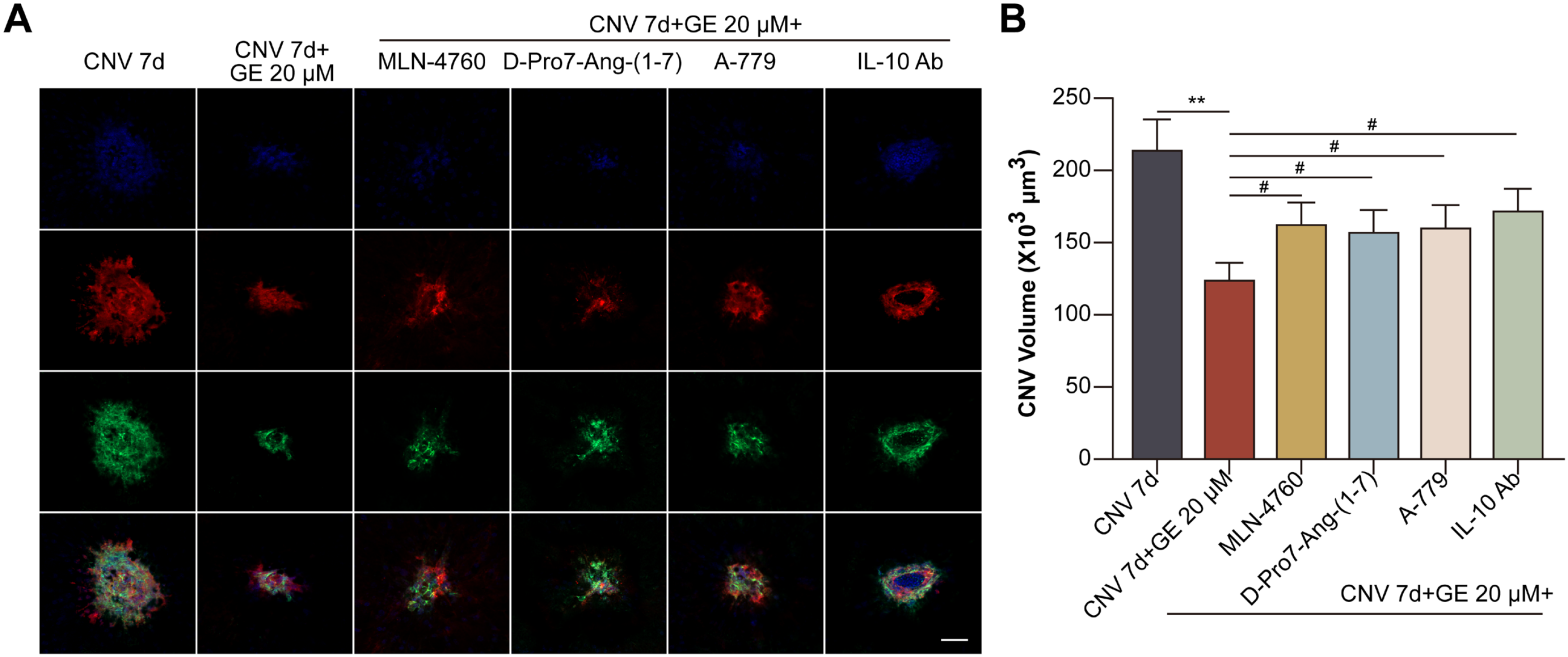
GE suppressed the development of CNV by activating the ACE2/Ang‐(1–7)/MasR/IL‐10 pathway in mice. Mice were divided into normal, CNV 7 d, CNV 7 d + GE (20 μM), CNV 7 d + GE + MLN‐4760 (10 nM), CNV 7 d + GE + D‐Pro7‐Ang‐(1–7) (100 nM), CNV 7 d + GE + A‐779 (100 nM), and CNV 7 d + GE + neutralising anti‐IL‐10 antibody (1 μg) groups. (A) Representative images of choroidal flat mounts after triple staining with anti‐IB4 antibody (a vascular endothelium marker; green), anti‐collagen IV antibody (a basal lamina collagen marker; red), and DAPI (a nuclear dye; blue) (scale bar = 100 μm). (B) CNV volume (^##^ *p* < 0.01 vs. the CNV 7 d group; ^%^ *p* < 0.05 vs. the CNV 7 d + GE group).

GE decreased the CNV volume, whereas MLN‐4760, D‐Pro7‐Ang‐(1–7), A‐779, or neutralising anti‐IL‐10 antibody partly counteracted the effects of GE on the CNV volume (Figure 7A,B).

### 2.5 GE Did Not Cause Ocular or Systemic Toxicity

**FIGURE 8:**
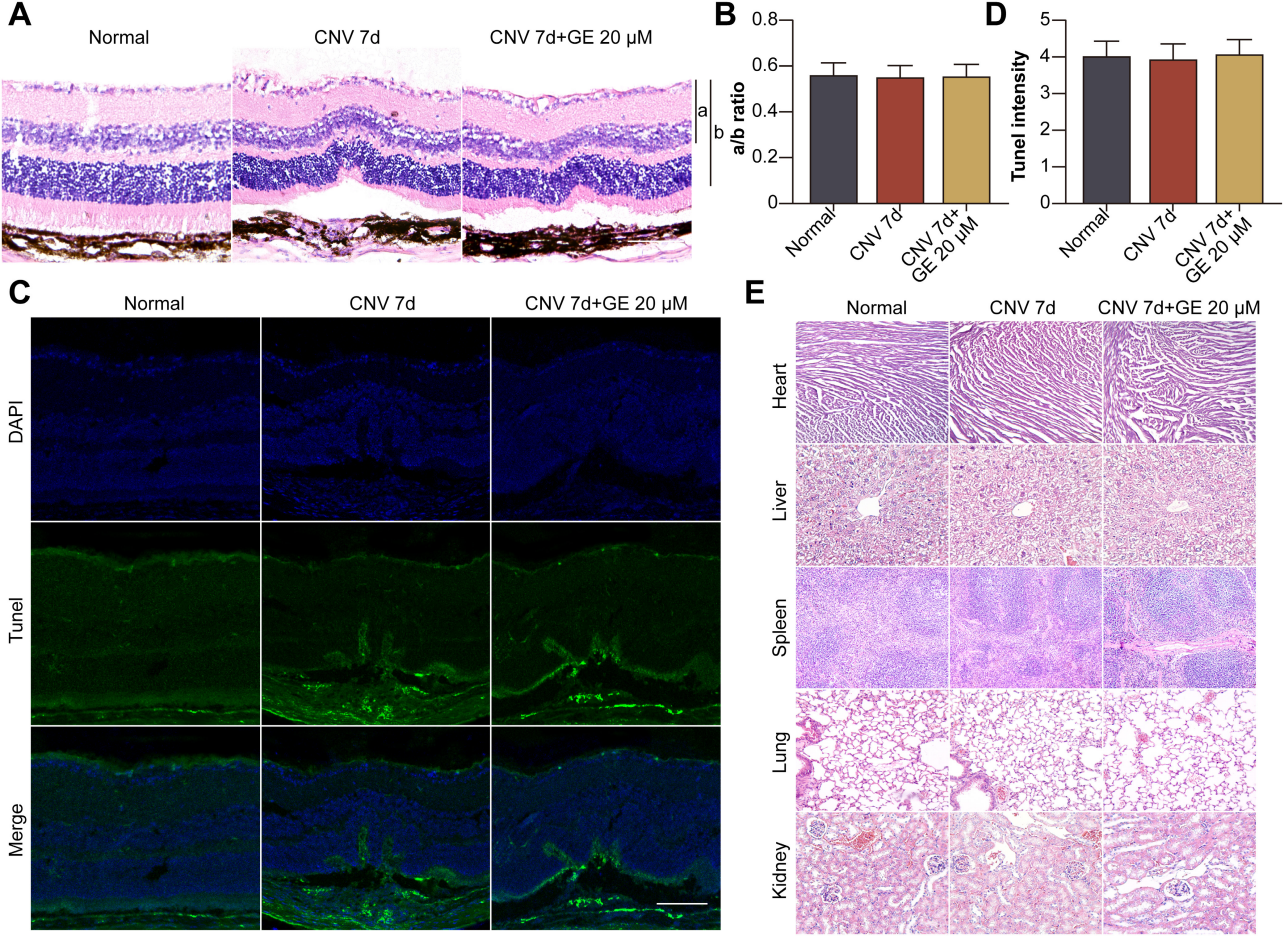
GE did not cause ocular or systemic toxicity. Mice were divided into normal, CNV 7 d, and CNV 7 d + GE (20 μM in 1 μL administered intravitreally on the same day of laser irradiation) groups. (A) Representative image of HE staining. (B) Quantification of the ratio of the distance from the ganglion cell layer to the outer edge of the inner nuclear layer (a) to the distance from the ganglion cell layer to the outer edge of the outer nuclear layer (b). (C) TUNEL (green) and DAPI (blue) staining of retina–RPE–choroid cryosections was performed to detect apoptotic cells (all scale bars = 50 μm). (D) The mean ratio of the number of TUNEL‐positive cells vs. the number of DAPI‐positive cells. (E) Representative images of HE staining of mouse heart, liver, spleen, lung, and kidney.

**FIGURE 9:**
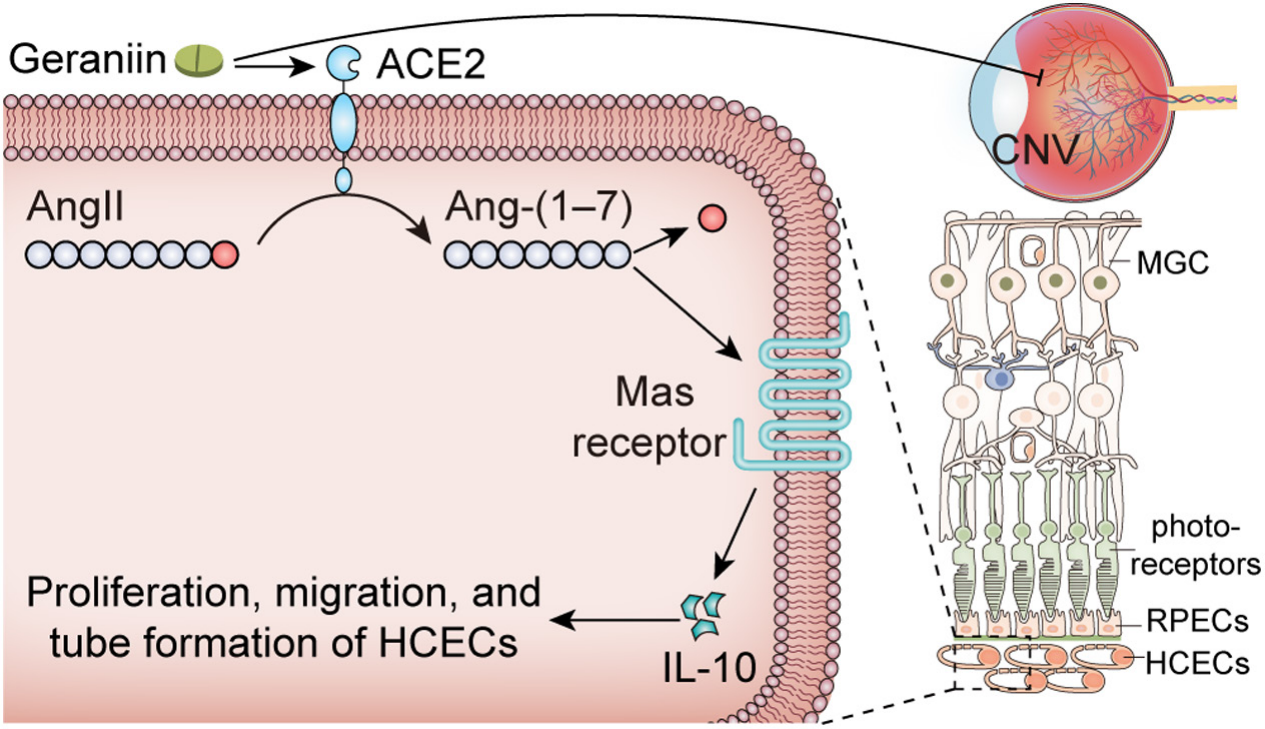
Role and mechanism of action of geraniin in CNV. Under hypoxic conditions, GE binds to ACE2 on HCECs, activating ACE2. Activated ACE2 catalyses the conversion of Ang‐II to Ang‐(1–7), which activates downstream MasR. Activation of the ACE2/Ang‐(1–7)/MasR pathway promotes the production of the anti‐angiogenic cytokine IL‐10 in CECs, thereby attenuating the proliferative, migratory, and tube‐forming abilities of HCECs. In addition, GE suppresses the development of laser‐induced CNV in mice.

GE did not cause morphological changes (Figure 8A,B) or cell apoptosis (Figure 8C,D) in retina–RPE–choroid tissues from mice with CNV. In addition, it did not affect the morphological features of vital organs, including the heart, liver, spleen, lung, and kidney, in mice with CNV (Figure 8E). Altogether, the results suggest that GE inhibits the development of CNV by activating the ACE2/Ang‐(1–7)/MasR/IL‐10 pathway in choroidal endothelial cells (Figure 9).

## 3 Discussion

In this study, we examined the effects of GE on hypoxia‐induced HCECs in vitro and mice with laser‐induced CNV in vivo. Under normoxic conditions, ACE2 was found to be expressed in HCECs but not in ARPE‐19 cells, which is consistent with the findings of a previous study [31], or retinal Müller cells. GE did not affect the mRNA or protein expression of ACE2 in HCECs under hypoxic conditions; however, GE at a dose of 40 μM increased the enzymatic activity of ACE2 in hypoxic HCECs.

GE promoted ACE2 to catalyse the conversion of Ang‐II to Ang‐(1–7) in hypoxic HCECs. Ang‐(1–7) subsequently increased the expression of downstream MasR, which promoted the production and release of IL‐10 from hypoxic HCECs. ACE2/Ang‐(1–7)/MasR pathway increases ERK phosphorylation and activation [32]. Of note, the activation of ERK pathway has been suggested to promote regulatory actions in enhancing the production of IL‐10 by human B cells via increasing the expression of transcript factor hypoxia‐inducible factor‐1α (HIF‐1α) [33], which was induced by hypoxic conditions in HCECs [34]. Therefore, we speculate that in hypoxic HCECs, ACE2/Ang‐(1–7)/MasR pathway promotes the activation of ERK pathway to elevate HIF‐1α, hence up‐regulates the production of IL‐10. IL‐10 released from local plasmacytoid dendritic cells accelerates the development of endometriosis via pathological angiogenesis during the early disease stage [35]. A study showed that compared with wild‐type mice, IL‐10^−/−^ mice showed a delayed neovascular response on day 6 after suturing, when approximately half of the cornea was neovascularised. This finding indicated that IL‐10 played a pro‐angiogenic role in suture‐induced corneal neovascularisation [36]. However, this study revealed that activation of the ACE2/Ang‐(1–7)/MasR/IL‐10 pathway by GE inhibited the proliferative, migratory, and tube‐forming abilities of hypoxic HCECs. This dual role of IL‐10 in angiogenesis may be attributed to the diversity of tissues and cells. The IL‐10 receptor (IL‐10R) is constitutively expressed on endothelial cells [37]. IL‐10 exerts its effects on regulatory T cells (Tregs) [38], macrophages [39], and B lymphocytes [40] in an autocrine manner. Therefore, we speculate that IL‐10 produced by HCECs binds to IL‐10R on HCECs to exert its effects.

Furthermore, GE activated the ACE2/Ang‐(1–7)/MasR/IL‐10 pathway in retina–RPE–choroid tissues from mice with CNV. The ACE2 inhibitor MLN‐4760, the Ang‐(1–7) antagonist D‐Pro7‐Ang‐(1–7), the MasR inhibitor A‐779, or neutralising anti‐IL‐10 antibody were used to differentiate the upstream and downstream relationship among ACE2, Ang‐(1–7), MasR, and IL‐10. Consistent with the results of in vitro experiments, in vivo experiments showed that ACE2 was produced by only CECs in mouse retina–RPE–choroid tissues. Studies have shown that macrophages [41] or microglia [42] may at least partly contribute to the production of IL‐10 in the retina–RPE–choroid complex in mice.

GE suppressed the development of CNV by activating the ACE2/Ang‐(1–7)/MasR/IL‐10 pathway and did not lead to ocular or systemic toxicity in mice. A study showed that the levels of inflammatory cytokines in the aqueous humour were higher in patients with CNV than in patients without other ocular pathologies undergoing cataract surgery [43], suggesting that inflammation can drive CNV. Based on this finding, trials have reported the use of intravitreal injection of glucocorticoids such as triamcinolone to inhibit inflammation in CNV [44]. Mice lacking nuclear factor erythroid 2‐related factor 2 (NRF2), a transcription factor that regulates oxidative stress response, show features of CNV [45], suggesting an important role of oxidative stress in the development of CNV. In addition to inhibiting angiogenesis, GE can alleviate inflammation [46] and oxidative stress [47]. The inflammatory response initiated by inflammatory microenvironment‐induced infiltrating neutrophils [24] and macrophages [48] contributes to the progression of CNV. GE inhibits an inflammation trigger lipopolysaccharide (LPS)‐induced infiltration of neutrophils and macrophages in the mouse injured lung [49]. However, the anti‐inflammatory and anti‐oxidative roles of GE warrant further investigation.

Until now, animal and cellular studies have revealed that GE possess bioactive properties such as anti‐inflammatory [50], anti‐viral [51], antioxidant [12], and anti‐hyperglycaemic [52]. In addition, GE shows no toxicities on animals [47] or cells [53]. These advantages support that GE has the potential for clinical translation. However, there are several challenges that should be addressed before GE can be seriously considered for clinical translation. Firstly, there is no thorough assessment about potential long‐term toxicities of GE intravitreal injection, which needs a long‐term administration of GE on animal CNV models. Next, the underlying mechanisms by which GE exerts anti‐angiogenic roles in CNV remain largely unclear and should be investigated in the future. Thirdly, there is also a large room for improvement to optimise drug delivery systems [54], such as nanoparticles and hydrogels.

In conclusion, this study reveals that GE alleviates CNV by activating the ACE2/Ang‐(1–7)/MasR/IL‐10 pathway in CECs, suggesting a novel treatment strategy for CNV. However, this study has some limitations that should be acknowledged. First, the role of anti‐angiogenic factors other than IL‐10, such as IL‐5 [55, 56] and IL‐13 [55, 57], in the protective effects of GE on CNV was not examined. Second, the optimal concentration of GE for its injection into the retina–RPE–choroid complex in mice with CNV was not identified. Last, fundus fluorescein angiography (FFA) and indocyanine green angiography (ICGA) were not performed to evaluate the CNV leakage volume and area in mice.

## Author Contributions

**Hongyi Lu:** conceptualization (equal), methodology (equal), project administration (equal), validation (equal), writing – original draft (equal). **Qi Cai:** data curation (equal), investigation (equal). **Lele Li:** software (equal), visualization (equal). **Jiayi Gu:** data curation (equal), methodology (equal). **Yuting Zhang:** data curation (equal), investigation (equal). **Haotian Sun:** investigation (equal), software (equal). **Hui Su:** supervision (equal), writing – review and editing (equal). **Lei Song:** funding acquisition (equal), supervision (equal), writing – review and editing (equal).

## Conflicts of Interest

The authors declare no conflicts of interest.

## Supporting information


**FIGURE S1.** GE alleviated the leakage and area of mouse laser‐induced CNV lesion in a concentration‐dependent manner. The mice were randomly divided into normal, CNV 7 d, CNV 7 d + GE 5 μM, CNV 7 d + GE 10 μM, and CNV 7 d + GE 20 μM groups. (A) FFA was performed to detect the leakage of CNV. (B) The leakage of CNV was analysed. (C) ICGA was performed to detect the area of CNV. (D) The area of CNV was analysed. *p < 0.05, **p < 0.01, ***p < 0.001 versus the CNV 7 d group. #*p* < 0.05, versus the CNV 7 d + GE 10 μM group.

## Data Availability

Data sharing not applicable to this article as no datasets were generated or analysed during the current study.
