# Supplementary figures and images for: Geraniin Alleviates Mouse Laser‐Induced Choroidal Neovascularisation by Inhibiting Choroidal Endothelial Cell ACE2/Ang‐(1–7)/MasR/IL‐10 Pathway
Source: J Cell Mol Med. 2024 Dec 2;28(23):e70228. doi: 10.1111/jcmm.70228 (PMC11611526; doi:10.1111/jcmm.70228)

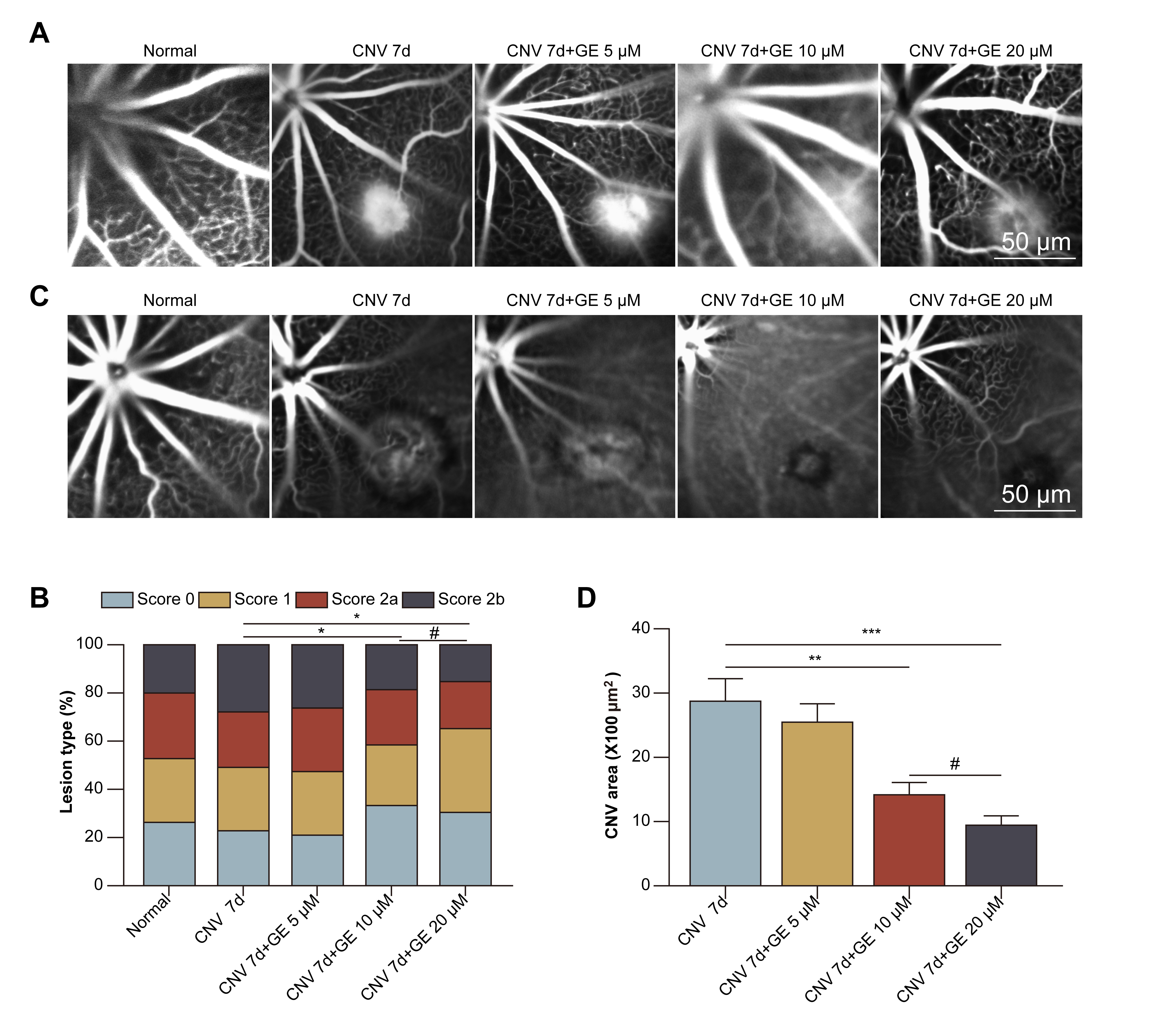

Supplement: Supplementary file 1 — FIGURE S1. GE alleviated the leakage and area of mouse laser‐induced CNV lesion in a concentration‐dependent manner. The mice were randomly divided into normal, CNV 7 d, CNV 7 d + GE 5 μM, CNV 7 d + GE 10 μM, and CNV 7 d + GE 20 μM groups. (A) FFA was performed to detect the leakage of CNV. (B) The leakage of CNV was analysed. (C) ICGA was performed to detect the area of CNV. (D) The area of CNV was analysed. *p < 0.05, **p < 0.01, ***p < 0.001 versus the CNV 7 d group. #p < 0.05, versus the CNV 7 d + GE 10 μM group. [file JCMM-28-e70228-s001.tif]
